# Case Report: Investigation and characterization of a multiple endocrine neoplasia type 1 case and its pedigree

**DOI:** 10.3389/fendo.2026.1747257

**Published:** 2026-01-23

**Authors:** Yifan Liu, Ling Cui, Shiwei Wang, Yanyan Chen, Ting Huang, Xin Hou

**Affiliations:** 1 Clinical Medicine Department, The Second Affiliated Hospital of China Medical University, Shenyang, Liaoning, China; 2 Department of Geriatric Endocrinology, The First Affiliated Hospital of China Medical University, Shenyang, Liaoning, China; 3 Department of Pathology, The First Affiliated Hospital of China Medical University, Shenyang, Liaoning, China

**Keywords:** c.512G>A variant, case report, genetic testing, multiple endocrine neoplasia type 1, pedigree

## Abstract

Multiple Endocrine Neoplasia Type 1 (MEN1) is an autosomal dominant inherited disease characterized by the combined occurrence of tumors in multiple endocrine organs. As a rare disease, the clinical manifestations of MEN1 are currently considered to be associated with the development of combinations of more than 20 different tumors, such as parathyroid adenomas, neuroendocrine tumors, pituitary tumors, as well as lipomas, thymic carcinoids, pheochromocytomas, adrenal adenomas, and ovarian tumors. However, the concurrent occurrence of MEN1 and teratoma is extremely rare in reported cases to date. Herein, we report a case of a female patient with MEN1 who was diagnosed with teratoma. Genetic testing identified the NM_130799.2: c.512G>A (p.Arg171Gln) variant, which was classified as a variant of uncertain significance (VUS). After extracting whole blood DNA from the patient and her relatives (7 individuals in total) for genetic testing, this mutation was found to be present in multiple members of the family, including some who were affected by MEN1. This finding suggests the potential pathogenicity of the mutation, although further research and longer-term follow-up are required to confirm this observation.

## Introduction

Multiple Endocrine Neoplasia Type 1 (MEN1) is an autosomal dominant hereditary disease typically characterized by the combined occurrence of tumors in multiple endocrine organs. As a rare disease, MEN1 has an incidence rate of 1 in 30, 000 to 1 in 50, 000, with an age of onset ranging from 6 to 81 years. Beyond the common clinical manifestations of parathyroid adenomas, neuroendocrine tumors, and pituitary tumors, its clinical presentations may also include more than 20 different tumor combinations, such as lipomas, thymic carcinoids, pheochromocytomas, adrenal adenomas, and ovarian tumors.

This disease is caused by mutations in the MEN1 gene. Localized to chromosome 11q13, the MEN1 gene spans approximately 9 kb and contains 10 exons. It encodes a 610-amino-acid protein known as multiple endocrine neoplasia protein (menin) (1). Menin has a molecular weight of 67 kDa (2), lacks homology with proteins in lower organisms (3), and is expressed in all tissues (4, 5). It plays a crucial role in regulating cell growth and maintaining cell cycle and genomic stability. Abnormal menin expression is a key driver of neuroendocrine tumorigenesis. Notably, among all tissues where menin regulates tumor development, neuroendocrine tissue is the only one with consistent menin mutations (6). Current research suggests that menin can function either as a tumor suppressor or an oncogenic initiator (7).

This case retrospective analysis focuses on the clinical characteristics and MEN1 gene mutation type of one patient with MEN1 and their family, who were diagnosed and treated in our hospital. The aim is to explore the relationship between the type of MEN1 gene mutation and the corresponding clinical manifestations through the study of the patient and their family.

## Case presentation

This study was approved by the Ethics Committee of the First Affiliated Hospital of China Medical University, and written informed consent was obtained from subjects.

**Figure 1:**
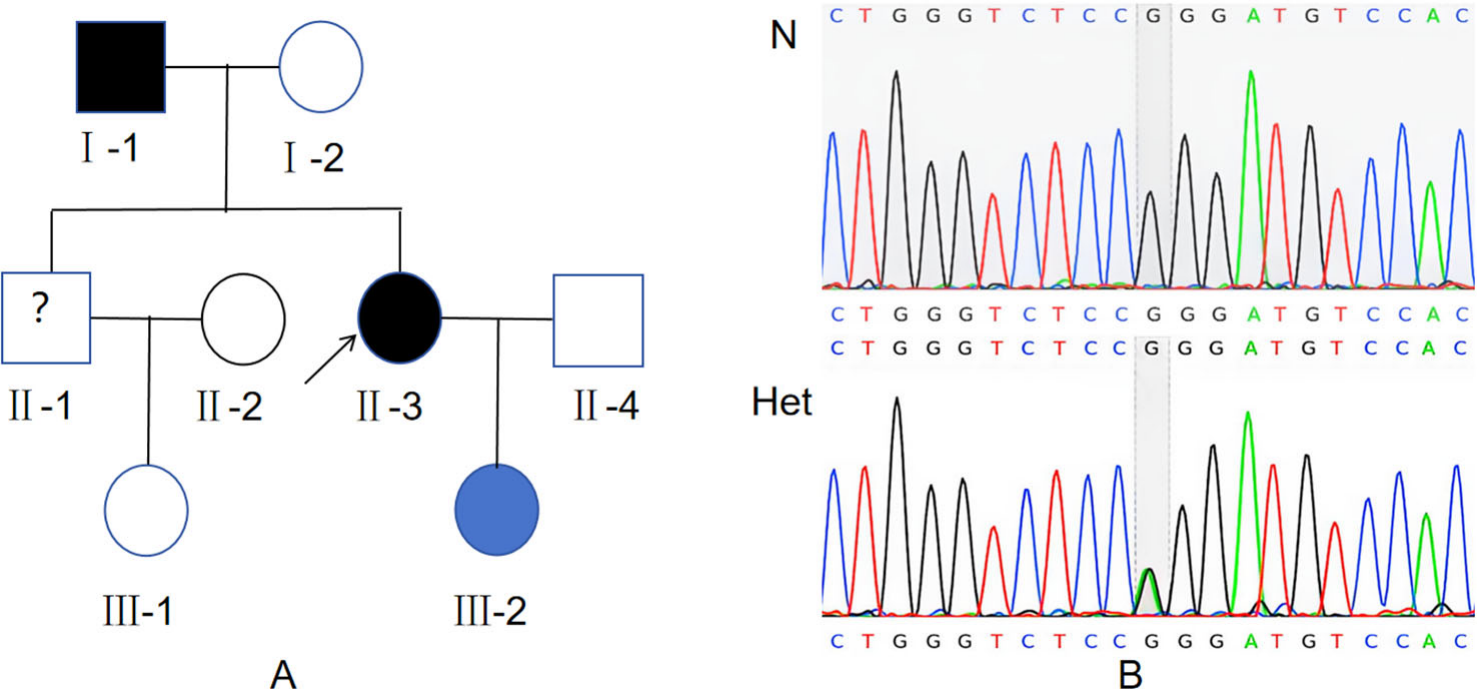
Identification of the heterozygous variant in the family. **(A)** Pedigree of the MEN1 Family. The individual indicated by the black arrow is the proband. Black symbols represent family members clinically diagnosed with Multiple Endocrine Neoplasia Type 1 (MEN1). Blue symbols represent carriers of the MEN1 mutation gene who have not yet received a clinical diagnosis. Blank symbols represent family members with no detected MEN1 gene mutation and no MEN1 diagnosis. Symbols marked with a question mark (?) represent family members who refused genetic testing. **(B)** Detection of a heterozygous mutation c.512G>A in exon 3 of MEN1 gene by Sanger sequencing.N: Wild type. Het: Heterozygote type.

**Figure 2:**
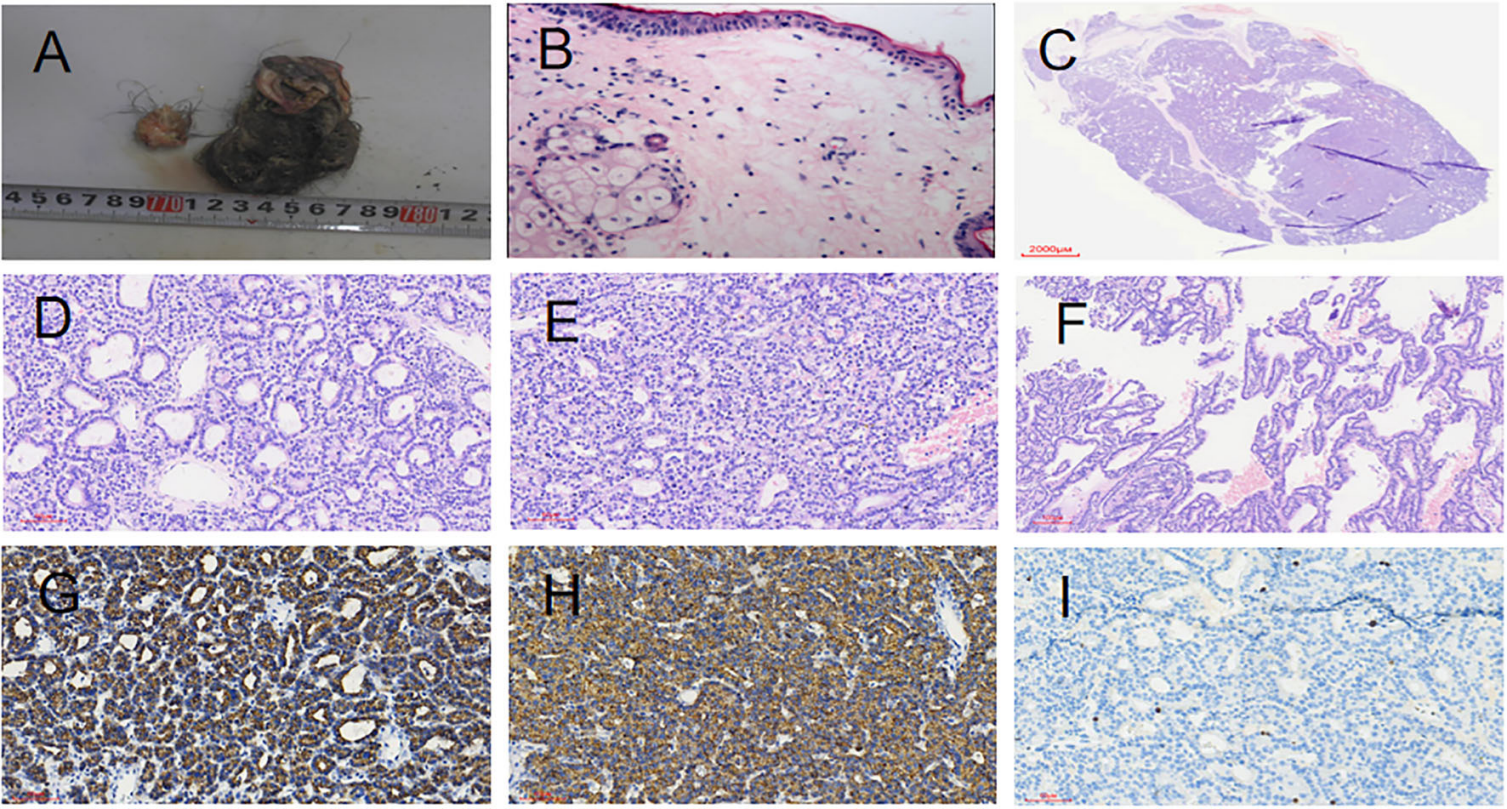
Gross findings of right ovarian teratoma **(A, B)**. **(A)** A pile of cystic wall-like tissue measuring 5 cm, with visible oil, hair, and a nodule-like structure of 2.2 cm. **(B)** HE staining (200×) showing squamous epithelium and skin appendages. Parathyroid Adenoma **(C-I)**. **(C)** Parathyroid adenoma. **(D)** Tumor chief cells arranged in an acinar pattern (HE staining, 200X). **(E)** Tumor chief cells arranged in a solid sheet pattern (HE staining, 200X). **(F)** Tumor chief cells arranged in a papillary pattern (100X). **(G)** Positive for PTH by immunohistochemical staining (200X). **(H)**: Positive for CgA by immunohistochemical staining (200X).**(I)**: Ki-67 proliferation index approximately 1% (200X).

Case 1: Proband (II-3, as shown in **Figure 1**), Female. At the age of 34, she sought medical attention due to recurrent right lower abdominal pain lasting for half a month. Transvaginal ultrasound of the uterus and adnexa revealed a 7.8 × 5.5 cm cystic mass in the right adnexal region, with a clear boundary and a liquid interior, accompanied by short linear hyperechoic streaks and hyperechoic mass reflections. The ultrasound suggested a cystic mass in the right adnexal region, with attention to the possibility of an ovarian cystic teratoma. Plain CT scan of the lower abdomen showed a thin-walled oval space-occupying lesion in the right adnexal region, measuring approximately 6.4 × 4.9 cm, with a clear boundary. Fat density and calcification density shadows were visible inside the lesion. The clinical diagnosis was a pelvic mass. The patient underwent laparoscopic enucleation of the right ovarian cyst. Postoperative paraffin pathology confirmed a right ovarian cystic mature teratoma (as shown in **Figure 2**).

At the age of 36, she consulted a doctor due to recurrent kidney stones for 2 years. Laboratory tests showed: Serum calcium: 2.87 mmol/L (reference range: 2.17-2.57 mmol/L), Serum phosphorus: 0.64 mmol/L (reference range: 0.81-1.52 mmol/L), Serum parathyroid hormone (PTH): 25.11 pmol/L (reference range: 0.66-12.00 pmol/L), 25-hydroxyvitamin D3: 10.38 ng/ml, Clinical diagnosis:primary hyperparathyroidism. The patient underwent resection of the right superior parathyroid adenoma. Postoperative pathology: Parathyroid adenoma with focal invasive growth (as shown in **Figure 2**). Postoperative laboratory tests showed: Serum calcium: 2.19 mmol/L (reference range: 2.17-2.57 mmol/L), Serum phosphorus: 1.17 mmol/L (reference range: 0.81-1.52 mmol/L), Serum PTH: 2.79 pmol/L (reference range: 0.66-12.00 pmol/L).

**Figure 3:**
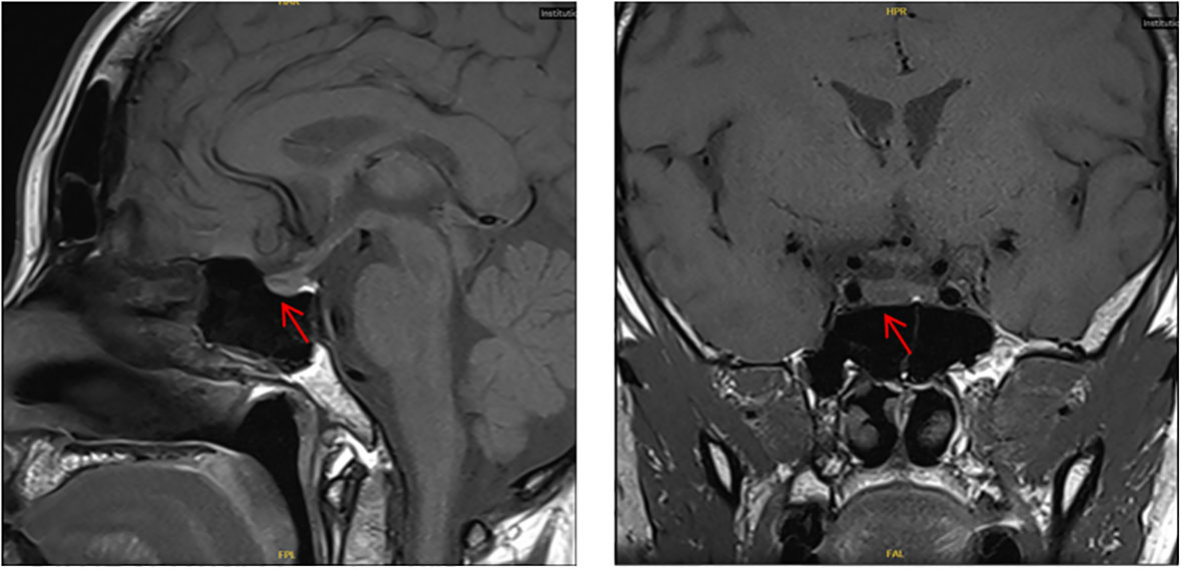
Case 1: Contrast-Enhanced MRI of the Pituitary Gland A hypointense nodular lesion is visible in the right lobe of the pituitary gland, consistent with a pituitary microadenoma.

At the age of 38, she sought medical attention due to dizziness. Pituitary contrast-enhanced MRI showed: a hypointense nodular lesion in the right lobe of the pituitary gland, with ill-defined boundaries, measuring 0.8 × 0.5 × 0.4 cm (left-right × anterior-posterior × superior-inferior); pituitary microadenoma was highly suspected (as shown in **Figure 3**). Laboratory tests for pituitary-related hormones revealed no abnormalities in Serum prolactin (PRL), growth hormone-releasing hormone (GHRH), luteinizing hormone (LH), follicle-stimulating hormone (FSH), thyroid stimulating hormone (TSH), corticotropin (ACTH) (**Supplementary Table S1** (8)). To evaluate the clinical suspicion of MEN1, the patient underwent whole-exome sequencing in 2019, which revealed no pathogenic mutations in the *MEN1* gene in the databases of variants associated with clinical phenotypes at that time.

In the same year, further thyroid ultrasound examination showed: bilateral thyroid nodules (C-TIRADS Category 3), with the larger nodule in the left lobe (C-TIRADS Category 4a). Breast ultrasound showed: multiple hypoechoic lesions in the right breast gland; the largest one was located at approximately the 10 o’clock position, measuring about 0.49 × 0.29 cm, with irregular shape and ill-defined edges, and no obvious blood flow was detected inside. Multiple hypoechoic lesions were also found in the left breast gland; the largest one was located at approximately the 2 o’clock position, measuring about 0.56 × 0.39 cm, with irregular shape and uneven edges, and no obvious blood flow was detected inside. Ultrasound diagnosis: bilateral breast hyperplastic nodules highly suspected (BI-RADS Category 3). Uterus and adnexa ultrasound showed: several nodular hypoechoic lesions in the uterine myometrium; the largest one was located in the posterior wall, measuring about 2.8 × 2.5 cm, with relatively clear outline and no blood flow detected. Ultrasound diagnosis: uterine fibroids.Pancreatic CT scan showed:No abnormalities of pancreatic lesions were identified.

**Figure 4:**
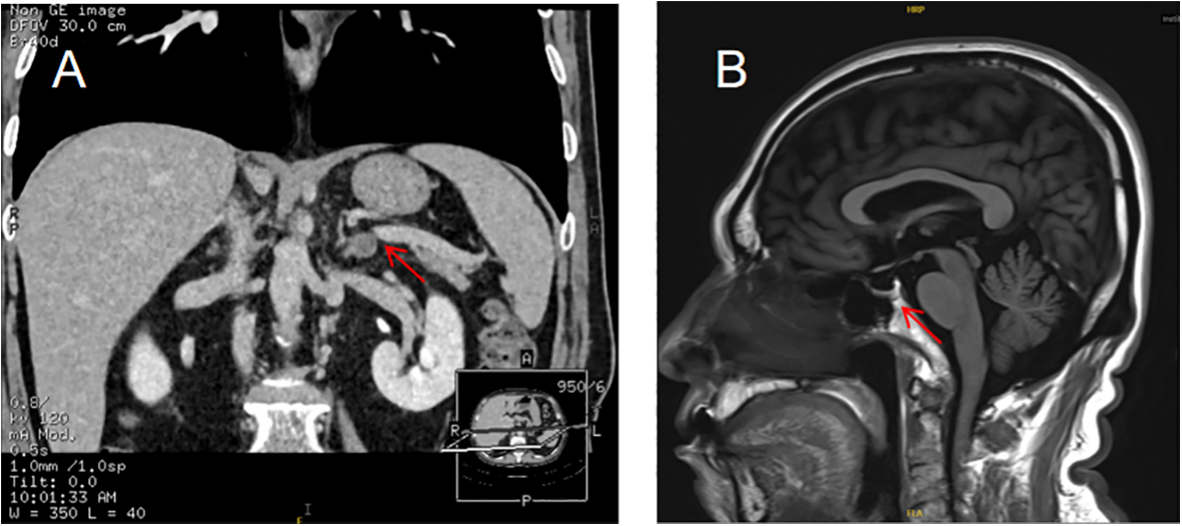
Case 2: Contrast-enhanced 3D-CT of the adrenal glands showed an oval nodule at the junction of the left adrenal gland, measuring 1.2×1.0 cm **(A)**. Cranial magnetic resonance imaging (MRI) showed empty sella turcica of the pituitary gland **(B)**.

Case 2: Proband’s Father (I-1, as shown in **Figure 1**), at the age of 68, presented to the hospital with a 18-year history of elevated blood pressure and a 1-year history of adrenal mass. Adrenal contrast-enhanced CT of the patient showed: a left adrenal mass (as shown in **Figure 4**). Combined with biochemical and imaging examinations, the possibility that this mass was caused by primary aldosteronism could not be ruled out (**Supplementary Tables S2**-**S4** (8)). Clinically, the diagnosis was made as “left adrenal mass (primary aldosteronism not excluded)”. The patient was given oral telmisartan 40 mg once daily and oral extended-release nifedipine tablets 20 mg once daily for antihypertensive treatment. During follow-up, serum potassium ion concentration was monitored, and no decrease in blood potassium has been observed so far. The patient had a subcutaneous lipoma in the left anterior chest.Esophagogastroduodenoscopy (EGD) showed: atrophic gastritis (Stage C3). Thyroid ultrasound showed: thyroid nodules (C-TIRADS Category 3), with the larger nodule in the left lobe (C-TIRADS Category 4a-). Brain MRI plain scan showed: empty sella turcica (as shown in **Figure 4**).The patient’s parathyroid hormone (PTH) level, serum calcium and phosphorus levels, and vitamin D level were all within the normal range. Additionally, no abnormalities were found in the gastrointestinal tract, and no kidney stones were detected.

With informed consent, our hospital collected whole blood samples for genetic testing from the patient and their relatives (a total of 7 individuals) on April 26, 2024. The samples were sent to Shenzhen Huada Medical Laboratory for family-based whole-genome sequencing using high-throughput sequencing technology. The genetic testing results indicated the detection of a variation in the MEN1 gene: NM_130799.2: c.512G>A (p.Arg171Gln)(as shown in **Figure 1**). The pedigree chart of this family is shown in **Figure 1**. Currently, other members of this family have no relevant clinical manifestations.

## Discussion

A diagnosis of MEN1 is of great significance to the patient’s family members. This is because the risk of developing the disease in the patient’s first-degree relatives is as high as 50%, and it is possible to determine whether they are carriers of the pathogenic gene through MEN1 gene mutation detection (9). In familial MEN1, the average age at diagnosis of the proband (the first family member diagnosed with the disease) is approximately 10 years older than that of relatives identified through screening (10). In addition, a study in Japan showed that there is a certain delay between the onset of initial symptoms and the diagnosis of MEN1, with an average latent period of 7 years (11). It took two genetic tests and nine years to reach a definitive diagnosis of MEN1 in this family’s proband. Therefore, it is particularly important to screen and diagnose MEN1 in advance through MEN1 genetic testing.

Genetic testing not only provides a convenient diagnostic tool for probands and their family members who carry the mutation, but also spares family members without the mutation from the psychological stress caused by lifelong clinical follow-ups. For the pedigree of this case, there is one individual with genetic variations (III-2) within the pedigree, but without clinical phenotypes. Up to now, this individual has been followed up, and she is 11 years old, with no clinical manifestations yet. We will continue to follow up this individuals.

Since the MEN1 gene was discovered in 1997 (1), research on the types of MEN1 gene mutations has been advancing toward completion, and various types of mutations have been identified and reported worldwide. However, current reports related to this specific variant are relatively rare (12–16). This variant had previously been reported only in Italy, Hungary, and Argentina, but not in China. Although genetic testing in 2019 did not report this mutation in the findings, this was primarily due to the limited research on variants associated with the clinical phenotype at that time. The relevant variant was ultimately detected through whole-genome sequencing of seven family members in 2024. According to the guidelines of the American College of Medical Genetics and Genomics (ACMG), this variant is classified as a variant of unknown significance (VUS) (criteria: PP2 + PP4 + BS1). A heterozygous mutation c.512G>A was detected in the sequence of exon 3 of the proband’s MEN1 gene. The variant p.Arg171Gln is a non-synonymous common variant, which is specifically characterized by a nucleotide substitution of G/A (rs607969) at position 64575505 on chromosome 11. This substitution results in a change from CGG to CAG in the codon, which further leads to an alteration in the amino acid sequence: the arginine (encoded by CGG) at position 171 is replaced by glutamine (encoded by CAG), denoted as R171Q. Existing studies indicate an inverse correlation between the R171Q allele frequency and the number of MEN1-related lesions (15). Given its similar prevalence across diverse populations and in a series of 100 tumors (both MEN1-related and unrelated), Lampichler K et al. hypothesized that R171Q does not directly drive MEN1-related tumorigenesis (15). However, it may still influence signal transduction cascades and other undefined MEN1-related biological processes and clinical phenotypes (13). In the present case, although no other pathogenic MEN1 variants have been identified, this variant should still be classified as a Variant of Uncertain Significance (VUS) due to the lack of relevant mechanistic studies.Meanwhile, given the limitations of the current research status, it remains possible that an unreported mutation contributes to the progression of the disease pathogenesis, rather than the c.512G>A (p.Arg171Gln) variant.

In addition to endocrine tumors, non-endocrine neoplasms associated with MEN1 have been documented, such as facial angiofibromas, collagenomas, and lipomas; there is also emerging evidence linking MEN1 to meningiomas and melanomas (24). Female patients with MEN1 also face an increased risk of developing breast cancer (17). Studies have shown that the menin protein can activate the estrogen receptor (ER), thereby enhancing susceptibility to hereditary breast cancer. Some variants identified in breast cancer tissues are similar to pathogenic variants that drive tumorigenesis—both lead to reduced stability of the menin protein and abnormalities in its interactions (18). As a top candidate gene specifically enriched in the body, the MEN1 gene regulates cytokine genes both *in vitro* and *in vivo (*19). Knockout of MEN1 has no effect on the *in vitro* proliferation or colony formation of various solid tumor cells (such as A549 and HCT116). However, it exerts a dual effect *in vivo*: In immunodeficient mice with an incomplete immune system, MEN1 knockout significantly promotes tumor growth. In immunocompetent mice, MEN1 knockout significantly inhibits tumor growth; this effect is consistent across multiple tumor models, including colon cancer (CT26), breast cancer (4T1), and pancreatic cancer (HKP1) (19). Meanwhile, even in the absence of other endocrine diseases or family history of MEN1, the presence of multinodular pancreatic neuroendocrine tumors in young patients may justify screening for MEN1 syndrome (12).

The patient’s clinical manifestations also included an ovarian mature cystic teratoma. Menin interacts with different proteins in various tissues and contexts; consequently, its functions exhibit high tissue and context specificity (20). In the context of cancer, depending on the tissue type and specific background, menin can act either as a tumor suppressor or a tumor initiator (7). In the ovarian tissue where the teratoma occurred in this case, menin is also increasingly recognized as a tumor promoter (7). Specifically, in ovarian cancer cells, menin regulates the transcription of genes associated with key cellular functions, including cell proliferation (21). It can thus be speculated that the MEN1 gene variation in this case may have played a role in the development of the patient’s ovarian mature cystic teratoma. A study conducted in the United States similarly confirmed that primary ovarian neuroendocrine tumors can arise in dermoid cysts (a type of ovarian teratoma) through MEN1-related mechanisms (22). Additionally, a case report from Italy also noted an association between MEN1 and teratomas (23). However, the patient in that Italian case was male, and the report focused on the relationship between mature testicular teratomas (classified as type II testicular germ cell tumors) and MEN1 gene mutations—this differs from the current case.Due to the lack of mechanistic research, these two entirely distinct diseases may still occur concurrently as dual primary tumors.

In summary, this case collectively underscores the critical role of family screening and genetic testing in diagnosing rare diseases. The c.512G>A variant maybe one of pathogenic mutation in the Chinese MEN1 population. Furthermore, it highlights that tumor surveillance in diagnosed MEN1 patients should be comprehensive, extending to atypical sites such as the ovaries to facilitate early tumor detection and improve overall prognosis.

## Data Availability

The original contributions presented in the study are included in the article/**Supplementary Material**. Further inquiries can be directed to the corresponding author.
