# Supplementary figures and images for: Case Report: Investigation and characterization of a multiple endocrine neoplasia type 1 case and its pedigree
Source: Front Endocrinol (Lausanne). 2026 Jan 23;17:1747257. doi: 10.3389/fendo.2026.1747257 (PMC12875917; doi:10.3389/fendo.2026.1747257)

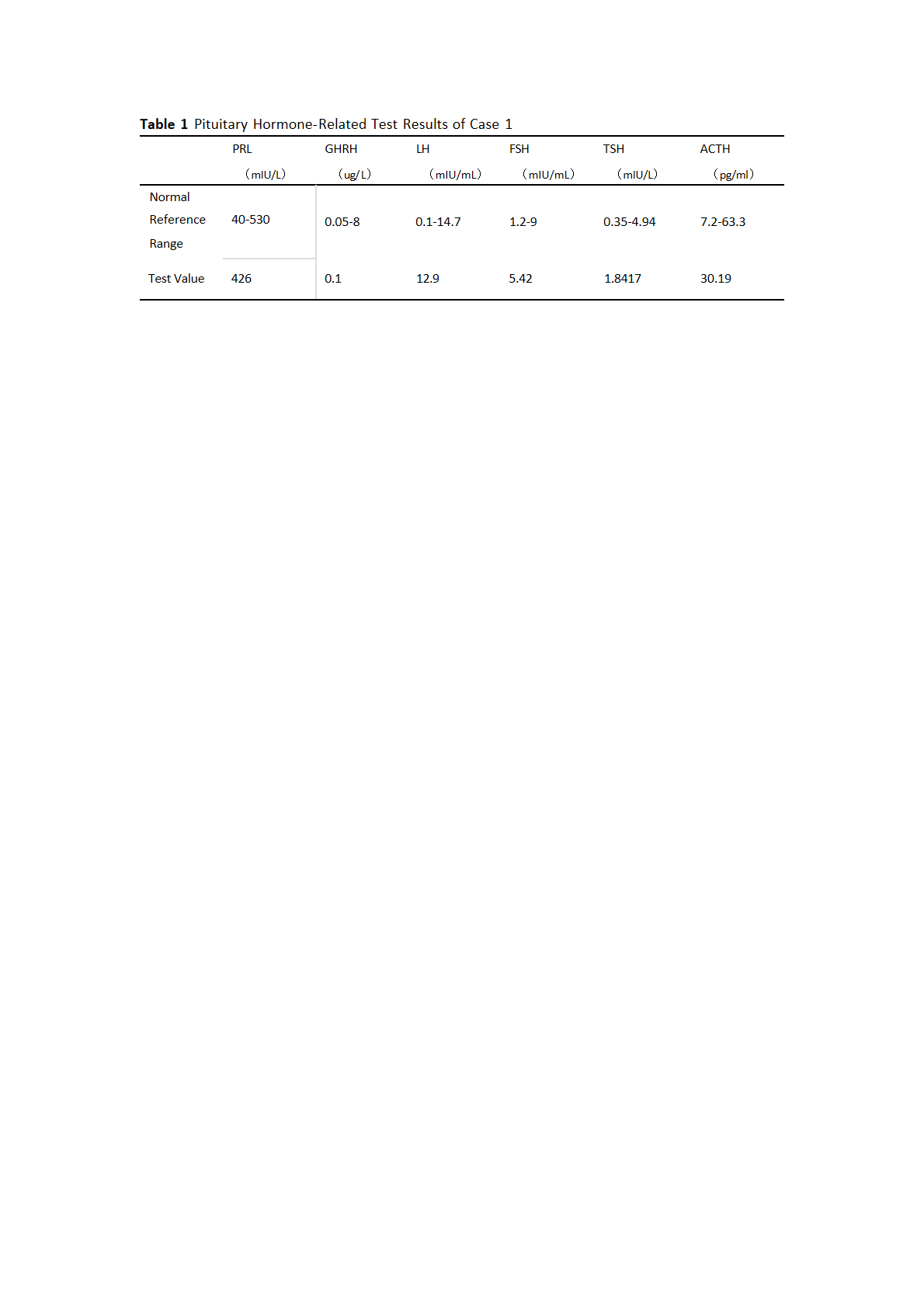

Supplement: Supplementary file 1 [file Image1.tif]

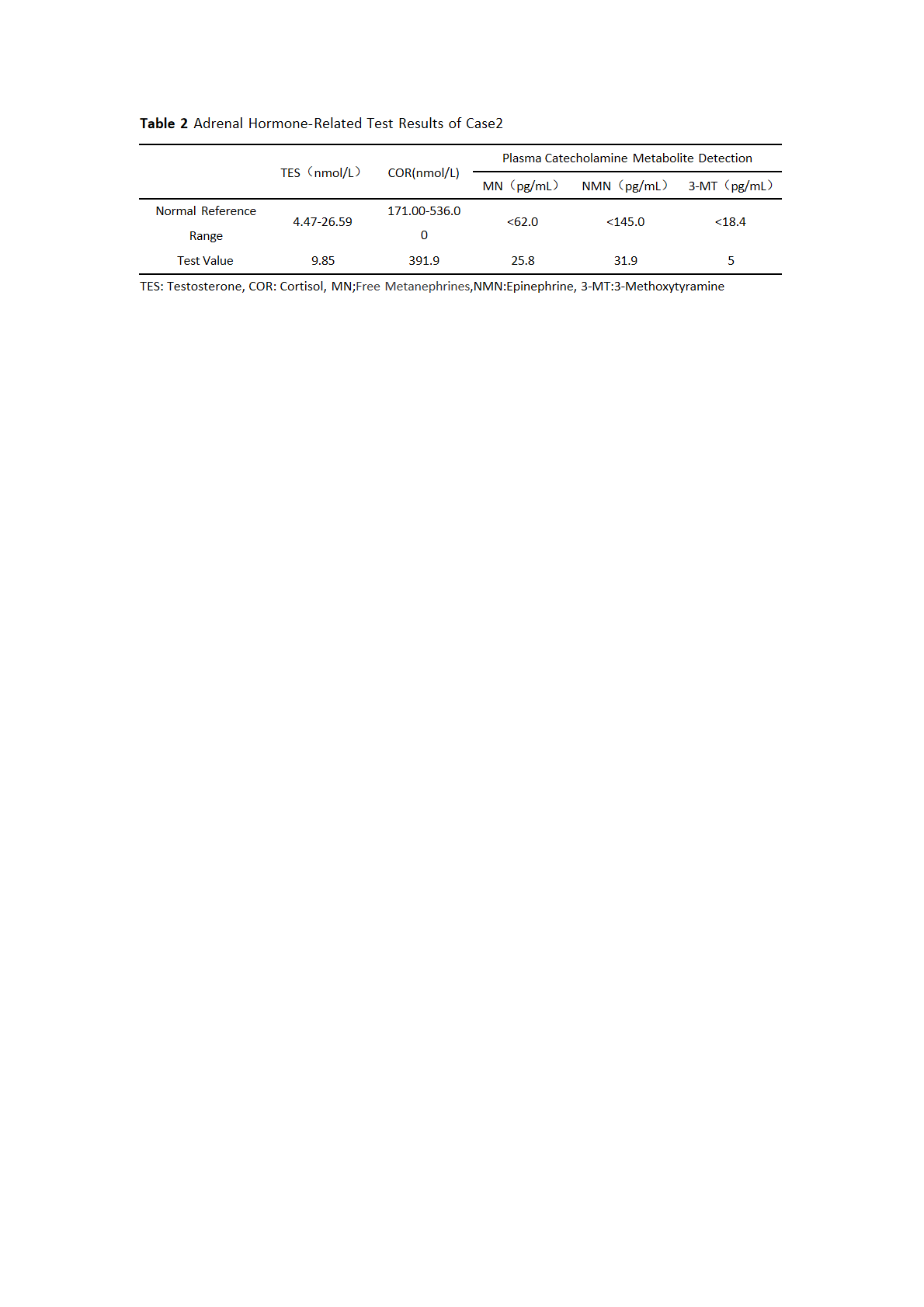

Supplement: Supplementary file 2 [file Image2.tif]

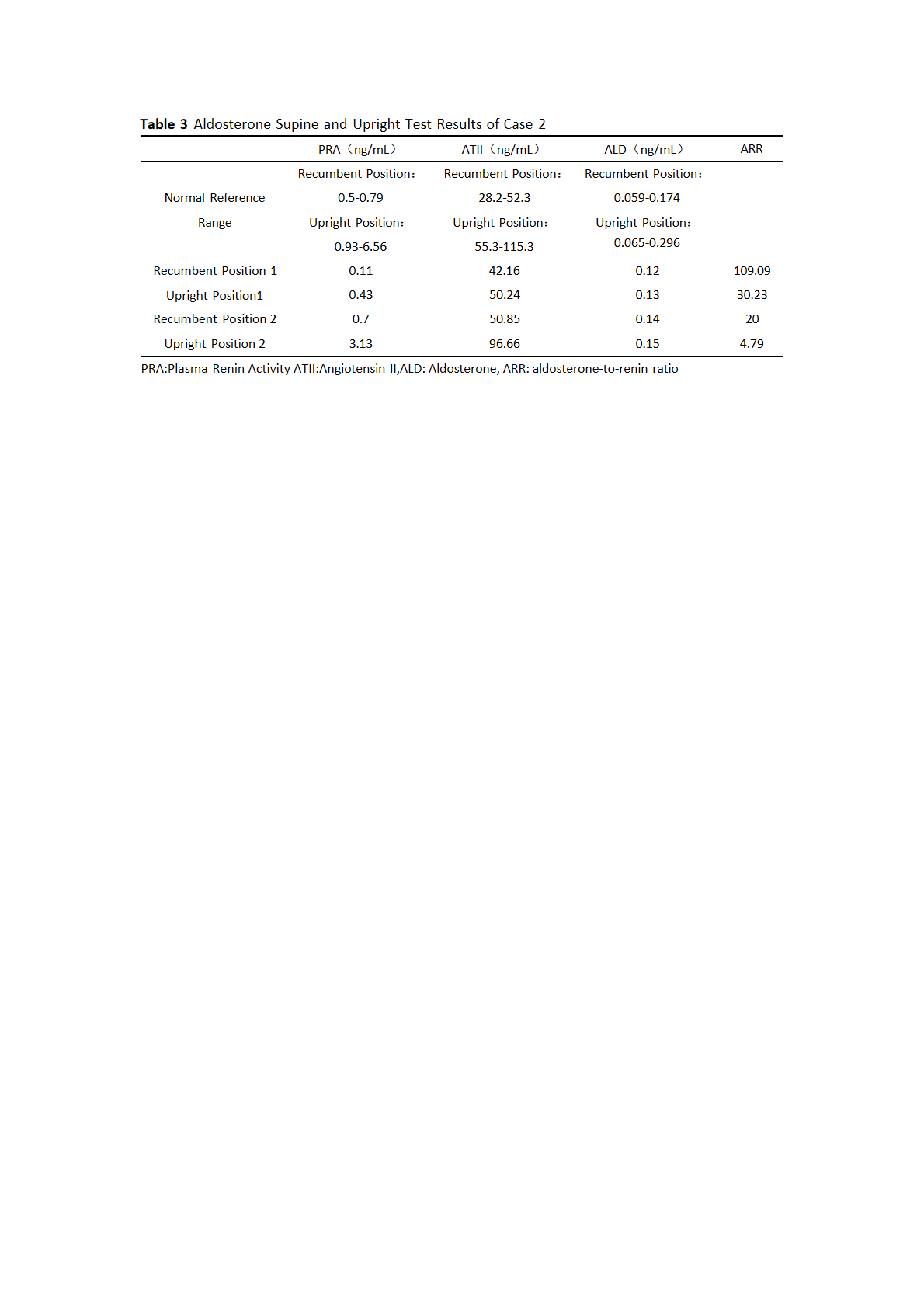

Supplement: Supplementary file 3 [file Image3.tif]

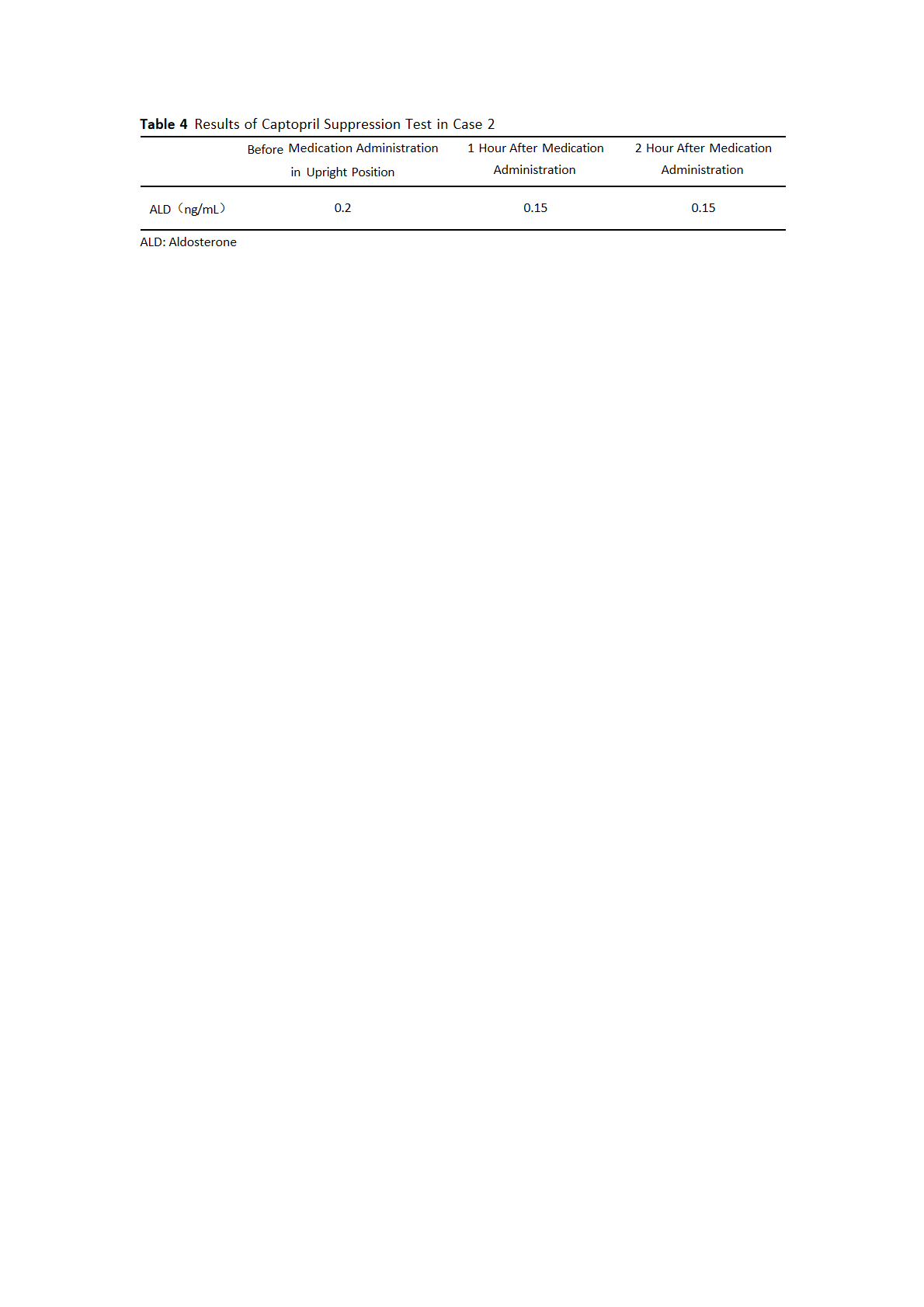

Supplement: Supplementary file 4 [file Image4.tif]
